# Rationale and protocol for the Time to Move Randomized Crossover Trial: morning versus evening time physical activity and CGM-assessed glucose levels in individuals with pregnancy hyperglycemia

**DOI:** 10.3389/fcdhc.2025.1709683

**Published:** 2026-01-13

**Authors:** Samantha F. Ehrlich, Bethany R. Hallenbeck, Jordan Lewis, Fatemeh Yousefi, John I. Miller, Nikki B. Zite, Kimberly B. Fortner, Walter W. Schoutko, Scott E. Crouter, Hollie Raynor, Jill M. Maples

**Affiliations:** 1 College of Education, Health, and Human Sciences, The University of Tennessee Knoxville, Knoxville, TN, United States; 2 Division of Research, Kaiser Permanente Northern California, Pleasanton, CA, United States; 3 The Department of Obstetrics and Gynecology, The University of Tennessee Health Science Center College of Medicine, Knoxville, TN, United States; 4 The High Performance & Scientific Computing Group, Office of Innovative Technologies, The University of Tennessee Knoxville, Knoxville, TN, United States; 5 The University of Tennessee Medical Center, Knoxville, TN, United States

**Keywords:** physical activity, time of day, glucose, hyperglycemia, diabetes, pregnancy

## Abstract

**Background:**

For most patients with pregnancy hyperglycemia, treatment includes lifestyle behavioral counseling for a healthy diet and physical activity (PA). Outside of pregnancy, emerging evidence suggests that the *timing* of PA (e.g., in the morning vs. evening) may modify its glucose-lowering effects. PA is an evidence-based, non-pharmacological strategy for managing glucose levels, and recommendations for PA *timing* could improve glucose levels in individuals with pregnancy hyperglycemia.

**Objective:**

To describe the rationale and protocol of the Time to Move Randomized Crossover Trial, which evaluates the effects of morning vs. evening PA on glucose levels across the 24-hour cycle.

**Methods:**

The eligibility criteria include singleton pregnancies in patients aged 18–40 years, identified as having gestational glucose intolerance [(GGI), a non-fasted, 50-g glucose challenge test, 1-hour value ≥130 mg/dl] or gestational diabetes mellitus [(GDM), by the one- or two-step procedure, at ≥24 weeks]. Participants who provide consent are randomized to first perform either morning PA (between 5 a.m. and 9 a.m., within 30 min–40 min of starting breakfast) or evening PA (between 4 p.m. and 8 p.m., within 30 min–40 min of starting dinner). All PA episodes consist of 30 min of moderate-intensity walking or stepping. Participants ultimately contribute 2 days in each of the three treatment conditions: morning PA, evening PA, and no PA, with one washout day between treatment conditions. Timestamped glucose measurements are obtained using Dexcom G6 or G7 continuous glucose monitors (CGM). The primary analysis will be intention-to-treat; per-protocol associations will also be explored. PA adherence is assessed using ActiGraph PA monitoring devices (i.e., the CentrePoint Insight Watch, worn on the non-dominant wrist), which provide continuous timestamped estimates of movement. Participants upload photos (i.e., in real time) of all foods and beverages consumed throughout the study period, and the timestamps of these photos are used to identify postprandial periods. One 24-hour dietary recall, aided by photo uploads, is also completed for each treatment condition.

**Conclusions:**

The Time to Move Randomized Crossover Trial addresses the gap in scientific knowledge regarding whether the *timing* of PA may be leveraged to maximize glucose control in individuals with pregnancy hyperglycemia.

**Clinical trial registration:**

## 1 Background

Gestational diabetes mellitus (GDM), or hyperglycemia first recognized during pregnancy, is diagnosed in up to 9% of pregnancies in the United States and has been increasing in recent years (1, 2). GDM confers an increased risk of adverse pregnancy outcomes, including premature delivery, induction of labor, Caesarean delivery, and delivery of a large-for-gestational-age neonate, as well as heightened risks for future cardiometabolic disease in the mother and obesity in the child. Similar risks have been reported in individuals with gestational glucose intolerance (GGI), who have abnormal screening test results for GDM without meeting the diagnostic criteria for GDM. Individuals with GGI are at an increased risk of delivering a large-for-gestational-age (LGA) neonate (3) and developing diabetes later in life (4).

For most patients with pregnancy hyperglycemia, treatment includes medical nutrition therapy for a healthy diet, physical activity (PA), and gestational weight gain within the ranges recommended for the patient’s pre-pregnancy body mass index (BMI) category, per the 2009 Institute of Medicine [IOM; now the National Academy of Medicine (2, 5)]. The recommended caloric intake and diet composition for individuals with pregnancy hyperglycemia do not differ from those for pregnant individuals without the condition (2, 5). They include a minimum of 175 g of carbohydrate (approximately 35% of a 2,000-calorie diet), a minimum of 71 g of protein, and 28 g of fiber (6–8). Behavioral counseling for a healthy diet, or Medical Nutrition Therapy (MNT) in a clinical setting, typically includes developing an individualized nutrition plan in conjunction with a Registered Dietitian Nutritionist (RDN) familiar with the management of GDM (2). Guidelines vary, but most recommend the distribution of carbohydrate intake into three small snacks (i.e., each 5%–10% of total carbohydrate intake) and three main meals [i.e., breakfast (10%–15% of total carbohydrate), lunch (20%–30%), and dinner (30%–40%)] per day (9). Lower carbohydrate intake is recommended at breakfast versus lunch and dinner due to the morning peak of cortisol secretion, which drives, at least in part, the high blood glucose values observed after breakfast (9) among individuals with pregnancy hyperglycemia.

Patients with pregnancy hyperglycemia are given the same standard recommendation for PA as healthy pregnant and non-pregnant adults: to achieve 150 min of moderate-intensity aerobic activity per week, preferably spread throughout the week (2, 5). A systematic review demonstrated improvements in glucose outcomes with exercise interventions (with exercise defined as PA that is intentionally performed for health or wellness) in pregnancy hyperglycemia; however, the review noted that heterogeneity in the types of activity (i.e., aerobic, resistance, or both), as well as the duration (i.e., 20 min–50 min per day) and frequency (i.e., 2–7 days per week) of activity, resulted in insufficient evidence to allow for recommending a specific type of program (2, 10). Walking is the most frequently reported type of PA among pregnant individuals (11). In pregnancies with GDM, postprandial walking (12), including light-intensity walking (13), effectively manages postprandial glucose excursions compared to no postprandial activity. More PA, specifically more steps accumulated in a day, is also associated with improved blood glucose concentrations in GDM (14). However, the distribution of the 30 min of walking, that is, whether to divide it into 10-minute episodes performed after each major meal or done continuously for a single 30-minute, postprandial episode, does not appear to differentially affect glucose outcomes (15, 16).

Meeting the PA recommendation through daily PA or participating in PA on most days of the week is recommended for non-pregnant adults with diabetes (17); however, no guidance on the *timing* of PA (e.g., morning or evening) has been provided. Outside pregnancy, emerging evidence suggests that the timing of PA may modify its impact on metabolic health. In a cross-sectional study of over 60,000 non-pregnant German adults, afternoon and evening activities provided greater metabolic health benefits than morning activity (18). PA recommendations for individuals with pregnancy hyperglycemia currently provide no guidance on the *timing* of PA (2, 5), although high blood glucose values earlier in the day have been reported in this population (9).

The timing of PA may also be leveraged to optimize glucose management and metabolic health in individuals with pregnancy hyperglycemia (19, 20). Behaviorally, performing PA at the same time each day helps with adherence by making PA planning easier and less complex; many behavioral PA interventions encourage action plans for PA, including plans for when PA will be performed (19, 20). PA timing may impact metabolism by affecting appetite, eating behaviors, and sleep (21, 22), in addition to physiological processes beyond PA-related increases in energy expenditure (19, 20), including greater fat oxidation (23) and various circadian influences [e.g., melatonin rhythms that influence sleep/wake timing (24–26)].

A scarcity of time to perform PA is a commonly cited barrier among non-pregnant and pregnant individuals alike (27); thus, in terms of adherence to PA, any time of day is technically the ‘right time’ for PA in this population. However, in the era of advanced technology and precision medicine, there is growing interest in leveraging rhythms, routines, and 24-hour behaviors to maximize metabolic health (28). PA is an evidence-based, non-pharmacological strategy for managing glucose levels. Following an acute bout of PA, glucose uptake remains elevated for approximately 2 h by insulin-independent mechanisms and for up to 48 h by insulin-dependent mechanisms, particularly if PA is prolonged (29).

This manuscript provides an overview of the Time to Move Randomized Crossover Trial. The Trial’s objective is to compare 30 min of moderate-intensity walking or stepping (30) (i.e., walking in place) under free living conditions, performed either in the morning (i.e., within 30 min to 40 min of starting breakfast) or in the evening (i.e., within 30 min to 40 min of starting after dinner). Previous research by our group suggests that stepping is an acceptable alternative to walking, and given the commonly cited barrier of a lack of time for PA, it may be advantageous in that it can be accomplished while performing other activities that are already part of the everyday routine (30). The primary outcomes include glucose metrics across the 24-hour cycle, assessed using continuous glucose monitors (CGM). We hypothesize that morning PA would improve daytime (6 a.m.–11:59 p.m.) glucose levels, as well as post-breakfast and post-lunch glucose levels, while evening PA will improve post-dinner, nighttime (12 a.m.–5:59 a.m.) (31), and pre-breakfast glucose levels, and thus ultimately improve 24-hour glucose levels. Secondary outcomes include mood during and sleep following bouts of PA. The timing of PA could potentially be leveraged to improve glucose management in this population, thereby leading to improved outcomes for mothers and children.

## 2 Methods

The Time to Move Randomized Crossover Trial is registered at ClinicalTrials.gov (ID# NCT06125704; first submitted on 30 October 2023, and first posted on 9 November 2023) and approved and monitored by the University of Tennessee Health Science Center College of Medicine, Knoxville, Institutional Review Board (Study #5079). To the extent possible, this methods paper follows the SPIRIT 2025 statement’s updated guidelines for protocols of randomized trials (32).

### 2.1 Study setting

The Time to Move study is being conducted at the University of Tennessee Medical Center (UTMC) in Knoxville, Tennessee. The UTMC serves as a referral center for a 28-county region in East Tennessee, Kentucky, Virginia, and North Carolina. In 2023, UTMC had 4,606 patient deliveries, of which 3.4% were GGI and 11.8% were GDM; 72.8% were White (non-Hispanic), 17.1% Hispanic, and 6.8% Black (non-Hispanic), and over half (57.6%) had public insurance (i.e., TennCare, the state of Tennessee’s Medicaid program). Pregnant individuals are screened for GDM using a 50-gram 1-hour oral glucose tolerance test (OGTT); if abnormal at the threshold of ≥130 mg/dl, it is followed by a 100-gram, 3-hour OGTT. GDM is diagnosed if two or more of the four plasma glucose values obtained during the 100-gram, 3-hour OGTT meet or exceed the Carpenter and Coustan thresholds (2, 33).

### 2.2 Eligibility and recruitment

The Time to Move study aims to randomize and follow 39 participants receiving prenatal care at UTMC. Recruitment began on 1 December 2023. The eligibility criteria include singleton, viable pregnancy with low suspicion of clinically significant abnormality or aneuploidy; 18–40 years of age (at the time of recruitment); comfortable communicating with study staff in English and completing study surveys in English (i.e., no translator needed); willingness to use a personal cell phone for study activities (e.g., complete surveys and telephone interviews); and GGI or GDM status. GGI is defined as a plasma glucose ≥130 mg/dL on the 50-g, 1-hour Glucose Challenge Test (GCT; non-fasted). Individuals with GGI undergo a 100-gram, 3-hour OGTT, performed after an overnight fast, at ≥24 weeks gestation, and GDM is diagnosed if at least two of the four plasma glucose values on the OGTT meet or exceed the following thresholds: 95 mg/dL for fasting, 180 mg/dL for 1-hour, 155 mg/dL for 2-hour, and 140 mg/dl for the 3-hour timepoint. Individuals with GDM diagnosed by the one-step procedure are also eligible [i.e., only a 75-g OGTT performed after an overnight fast, at ≥24 weeks gestation, with at least one plasma glucose value meeting or exceeding the thresholds of 92 mg/dL for fasting, 180 mg/dL for 1-hour, and 153 mg/dL for the 2-hour timepoint]. Individuals with GDM identified by a 50-g, 1-hour GCT plasma glucose ≥180 mg/dL are also eligible.

The exclusion criteria are as follows: physician determination that PA, particularly walking, should be limited in the current pregnancy; a prior diagnosis of diabetes, outside of pregnancy; current use of daily medications known to alter insulin resistance and/or metabolic profiles (e.g., insulin, metformin, corticosteroids, and anti-psychotics); current use of medication for polycystic ovarian syndrome (PCOS); and conditions that interfere with one’s ability to follow a strict, time-based study protocol (e.g., psychiatric illness). Individuals working night shifts or with circumstances (e.g., lifestyle factors, religious observance) that precluded their ability to follow a strict, time-based study protocol are also excluded.

Potentially eligible patients are identified by clinic and study staff, who review the electronic medical records to conduct a preliminary eligibility assessment. The study staff then briefly explain the study, either in-person (i.e., meeting the patient at their OGTT) or by phone or text message, to assess interest. Potentially eligible patients, per the preliminary assessment, are then reviewed by an MD for the final determination of eligibility.

### 2.3 Consent and randomization

Potential participants are invited to schedule an Information Session (remote or in-person) to learn more about the study. They are emailed a link to the study consent form and asked to review it ahead of the Information Session. The consent and all study procedures are reviewed at the Information Session; time is allotted to answer questions prior to signing the consent.

**Figure 1:**
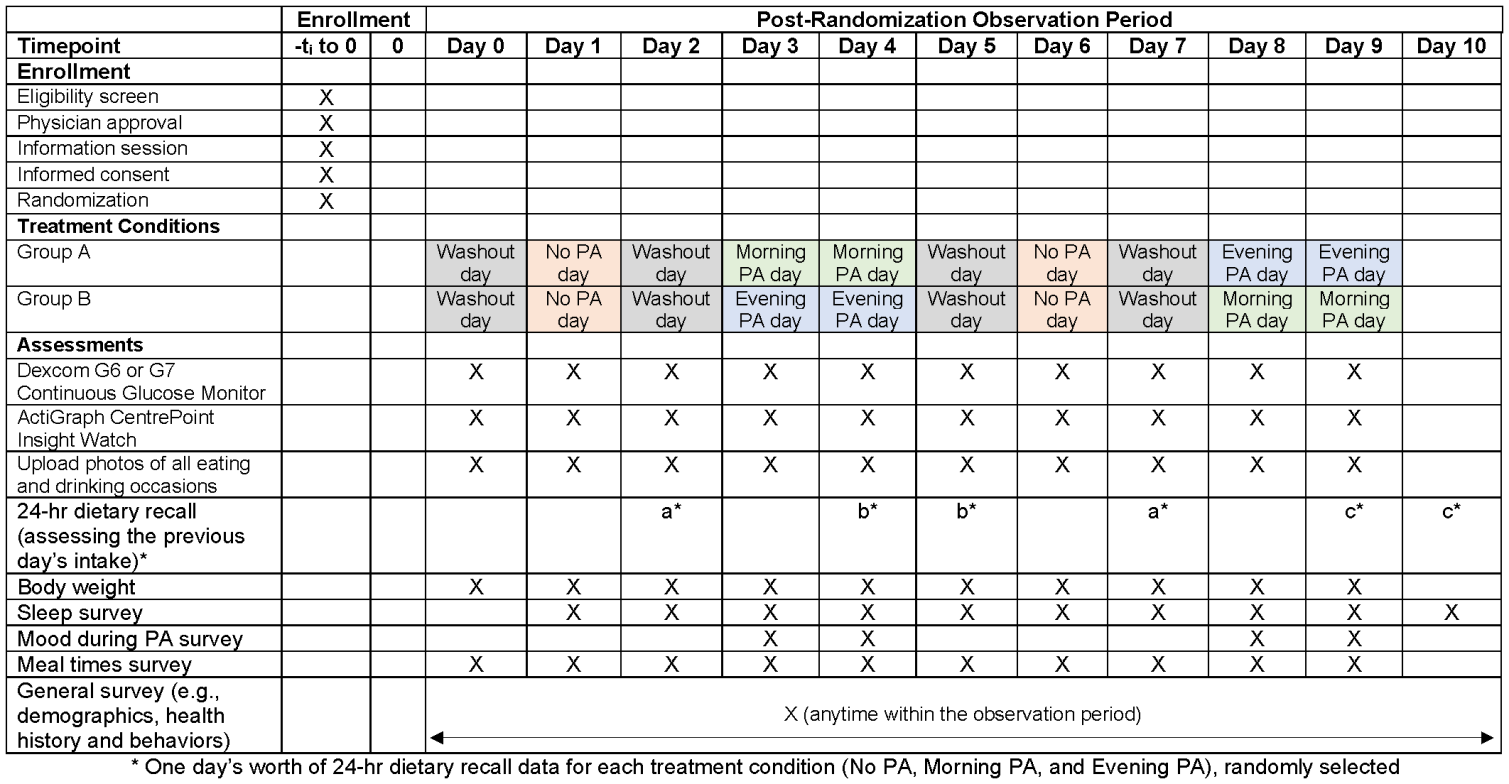
SPIRIT 2025 diagram of the schedule of enrollment, interventions, and assessments for the Time to Move Randomized Crossover Trial, the University of Tennessee Medical Center, Knoxville, Tennessee, USA.

Participants are then randomized in SAS to either morning PA followed by evening PA or evening PA followed by morning PA (**Figure 1**, [*18*]); the crossover design allows each participant to serve as their own control. Participants are instructed to perform no PA for health or wellness on the day of the Study Visit (i.e., day 0) and on days 2, 5, and 7, which served as wash-out days. PA (described in greater detail in the following section) is performed on days 3 and 4 and again on days 8 and 9. No PA for health or wellness is performed on days 1 and 6, thereby providing two full days’ (i.e., 48 h) worth of data under each treatment condition (i.e., morning PA, evening PA, and no PA).

Randomization is blocked (block size 4) and stratified by GGI and GDM. The allocation is concealed by a colleague who is not a member of the study team. The study investigators are blinded to treatment assignment: the analytic programmer will present all results by treatment A, B, and C until the conclusion of the trial (i.e., the investigators will remain blinded through the drafting of the study’s *Results* section and then be unblinded to write the *Discussion* and finalize the study).

### 2.4 Conditions

For PA, participants are instructed to perform 30 min of moderate-intensity walking or stepping (i.e., walking in place or around a small area) (34) following the respective meal. Moderate-intensity PA is described as activities during which you can comfortably talk but not sing. Moderate-intensity walking or stepping is described as a cadence of approximately 100 steps per minute. Well-known songs at 100 beats per minute, such as the Bee Gee’s *Stayin Alive* and Beyonce’s *Crazy in Love*, are recommended to help participants walk or step at 100 steps per minute (35). On days they are assigned morning PA, participants are instructed to walk or step between 5 a.m. and 9 a.m. and to begin their PA 30 min–40 min after the start of their breakfast. On days they are assigned evening PA, they are to walk or step between 4 p.m. and 8 p.m. and begin 30 min–40 min after the start of their dinner. Adherence to PA is defined as completing at least 80% of the assigned PA during the assigned window (i.e., 24 min or more of the continuous walk/run activity type, as detected by the device (see Section *Device-assessed physical activity*), during the assigned timeframe). Participants are instructed to not perform any additional PA intentionally for health or wellness outside of the assigned (by day and time range) walking or stepping for the duration of the study.

A personalized calendar outlining each day’s PA (or no PA) assignment and data collection activities (described below) and their time of day in a checklist format is provided at the Study Visit. The study staff also review a handout on ‘Keeping it safe’ during PA in pregnancy with the participant and develop an Action Plan for their PA. The Action Plan includes specific details on the times they will perform their PA (within the assigned ranges/days), the location(s) where they will perform their PA, the type of PA (i.e., walking or stepping), a strategy for maintaining PA at least a moderate intensity throughout their sessions (e.g., using either the ‘talk test’ or walking to a 100 beats per minute song), and potentially, with whom they will do their PA or what they will listen to/watch during their sessions. Participants are offered personalized encouraging text message reminders for their PA. To the extent possible, roles at the Study Visit are split: one member of the study team works with the participant on their PA Action Plan and another member of the team reviews the data collection procedures.

### 2.5 Data collection

Data are collected by trained study staff at the Study Visits (e.g., weight using a wireless WYZE scale and height using a Seca stadiometer) and abstracted from the electronic health records (i.e., pre-pregnancy BMI, estimated delivery date based on ultrasound, delivery date). Additional data are obtained from the CGM and wrist-worn PA monitoring device worn by participants, study e-surveys, an electronic scale (for body weight upon waking each day of the study period), and dietary recall telephone interviews to assess eating behavior for one 24-hour period under each treatment (morning PA, evening PA, and no PA). Protected health information is removed from all analytic datasets, and the data sources linked by study ID number. Protected health information required for the conduct of the trial is stored on password-protected servers housed at the University of Tennessee Knoxville and the the University of Tennessee Health Science Center College of Medicine, Knoxville. The de-identified data are pre-processed to make feature types consistent across data sources and to harmonize the multiple data elements.

#### 2.5.1 Glucose outcomes

Initially, the participants wore blinded Dexcom G6 CGMs. On 9 December 2024, the IRB approved a modification for unblinded Dexcom G7 CGMs due to the phasing out of the G6. The CGM is placed on the back of the upper arm. The participant is asked to use the side on which they were least likely to sleep for CGM placement (i.e., to avoid compression lows). All CGM data are accessed via Dexcom’s Clarity system.

Glucose outcomes include the mean, area under the curve (AUC), and coefficient of variation (CV) for 24-hour glucose, daytime glucose, and nighttime glucose; percentage of time in range (TIR), percentage of time above range (TAR), and percentage time below range (TBR) were added after the trial was underway. For 24-hour glucose, the start times will vary by treatment; specifically, 24-hour summaries are anchored (begin at) at 4 a.m. for morning PA days, 3 p.m. for evening PA days, and midnight on no PA days. The daytime window is defined as 6 a.m. to 11:59 p.m., and the nighttime window as 12 a.m. to 5:59 a.m (31). Glucose outcomes also include the mean, AUC, and CV for the pre-breakfast (calculated for the 120 min prior to the start of breakfast) and all post-meal periods (calculated for the 120 min after the start of breakfast, lunch, and dinner). The identification of the *time* of the start of breakfast, lunch, and dinner is described in Section *Dietary intake and meal times*.

#### 2.5.2 Device data processing and cleaning

Physical activity and sleep are assessed using ActiGraph’s (now Ametris’) CentrePoint Insight Watch. The Watch data are downloaded from ActiGraph’s (now Ametris) CentrePoint system, a cloud-based platform designed to manage and analyze physical activity and sleep data. The de-identified raw (32 Hz sampling rate) device data are exported as 1-second epochs and raw CSV files and read into RStudio. The device sensor data are first examined for wake and sleep times. Sleep time and non-sleep time are defined using participant sleep diaries. Next, the non-wear time is identified using the Choi algorithm (36–38). Days with less than 600 min of non-sleeping wear time will be excluded from the analyses.

#### 2.5.3 Device-assessed physical activity

Wrist-specific two-regression algorithms are used to calculate the metabolic equivalent of task (MET) and coefficients of variation (CV) for each time-stamped 1-second, non-sleep period (39). MET values are used to define movement intensity as sedentary (≤1.50 MET), light (1.51 MET–2.99 MET), moderate (3.00 MET–5.99 MET), or vigorous (≥6.0 MET), and the 1-second periods are then collapsed to the minute level (i.e., the average MET value for each minute determined). The total minutes spent in sedentary, light, moderate, and vigorous intensity activity are summed at the day level and for the periods used to categorize the glucose data (i.e., daytime, nighttime, for 120 min before the start of breakfast, and for 120 min after the start of the breakfast, lunch, and dinner).

The data are also used to estimate the activity type, which is designated as continuous walk/run, intermittent, or sedentary activity. First, the MET values assigned to each 1-second non-sleep period are used to identify sedentary activity (≤1.50 MET). Then, 1-second non-sleep, non-sedentary periods with CV ≤21.2% for devices worn on the right wrist or CV ≤19.4% for devices worn on the left wrist are designated as continuous walk/run activity, and those with CV >21.2% for devices worn on the right wrist or CV >19.4% for devices worn on the left wrist are designated as intermittent activity. Continuous walking/running activity is further classified as light-, moderate-, or vigorous-intensity (39). The total minutes of continuous walking/running activity is then summed for the day level, and the periods used to categorize the glucose data.

#### 2.5.4 Device-assessed sleep

After the start of the trial, device-assessed sleep metrics were added as secondary outcomes. After identifying “sleep times” from the self-reported diaries, the Cole–Kripke sleep algorithm (40) is applied to obtain sleep metrics that included sleep duration, sleep efficiency (defined as the total percentage of algorithm-detected sleep within the diary-defined sleep window), total waking time during the sleep window, and nap duration.

#### 2.5.5 Study e-surveys

Study data are collected and managed using the REDCap electronic data capture tools hosted at the University of Tennessee Health Science Center. Each morning, participants receive a text message with a link to a *Daily Sleep Survey* to complete upon waking. The *Daily Sleep Survey* includes a modified version of the Pittsburgh Sleep Quality Index [PSQI (41)] with questions pertaining specifically to sleep the night before (as opposed to usual sleep habits during the past month, as in the original PSQI). The *Daily Sleep Survey* assesses the time they went to bed the night before, the time they fell asleep, the time they woke up for the day, the number of times they woke up during the night (e.g., to go to the bathroom), the quality of their sleep the night before (i.e., very poor, poor, fair, good, or very good), use of medication to help with sleep, the presence of a bed partner, whether they had trouble sleeping, and the reason they had trouble sleeping (i.e., could not get to sleep within 30 min, had to get up to use the bathroom, could not breathe comfortable, coughed or snored loudly, felt too cold, felt too hot, had pain, other—please describe). The *Daily Sleep Survey* also assesses (i.e., for the day before), napping, whether or not they completed their assigned PA, periods when the CentrePoint Insight Watch was removed, and whether they experienced an injury or accident. *A priori* metrics of interest included the duration (length in hours and minutes) of sleep, sleep efficiency (percentage of time spent asleep while in bed), and sleep quality (i.e., very poor, poor, fair, good, or very good).

Participants additionally complete a *Daily Meal Times Survey* each evening to collect data on the exact times they started breakfast, lunch, and dinner that day.

On the days PA is assigned, participants are texted a link to a *PA Mood Survey* and complete it within 2 h of completing their PA. The *PA Mood Survey* includes questions on the circumstances of PA (e.g., walking, stepping, or a combination of the two; outside, indoors, or both; while listening to music or watching a video), as well as their self-reported mood state during PA. Mood state is assessed using a short version of the Multi-Dimensional Mood Questionnaire (42). Specifically, questions on the extremes of energetic arousal (43), which range from energetic (positive) to worn-out (negative), are assessed using a 6-point Likert scale. Two items assess the extremes of the energetic arousal scale (i.e., during my walking, I felt “energetic” or “worn-out,” each on a 6-point scale representing “definitely not” to “extremely”).

To aid with adherence and minimize recall times and bias, at the Study Visit, participants are asked for their preferred times, within the study’s prescribed window, to complete the *Daily Sleep Survey*, *Daily Meal Times Survey*, and the *PA Mood Surveys*, and the survey schedule is set up accordingly.

At the beginning of the study period, participants are emailed a link to an asynchronous *General Survey*. Participants have the entire study period to complete the *General Survey* (i.e., they can stop at any point and pick back up where they left off at a later time). The *General Survey* assesses demographic characteristics, medical and reproductive history, symptoms of depression (i.e., using the Patient Health Questionnaire (PHQ-9), but excluding the item assessing suicide ideation (44)), the Risk Perception Survey for Developing Diabetes (RPS-DD (45)), and health behaviors, including PA during the month before joining the study, assessed with the Stanford Leisure Time PA Questionnaire (46, 47) and the Pregnancy Physical Activity Questionnaire (48, 49). Smoking and alcohol consumption, and the safety and walkability of their neighborhood are also assessed. Finally, the *General Survey* includes several items pertaining to their preferred time for physical exercise and general circadian preference (i.e., morning vs. evening type) from the Morningness–Eveningness Questionnaire [MEQ (50, 51)].

#### 2.5.6 Dietary intake and meal times

Dietary intake is assessed using three 24-hour dietary recalls (24HR) using the United States Department of Agriculture (USDA) five-step multiple pass method (MPM) (52). One 24HR is completed for each treatment condition. Specifically, one of the two days assigned to each treatment condition is randomly selected to determine the 24HR observation day. Participants are called the following day and asked to describe all foods and beverages consumed the previous day. To aid with adherence to the 24HR calls, the activity is highlighted on the participant’s personalized study calendar, and they are asked for their preferred time to conduct the 24HR assessment.

Before beginning the 24HR, a trained research assistant explains the MPM and then obtains the recall data. Two tools are used to enhance the accuracy of self-reported dietary intake data. First, the participants receive a hard copy of a Food Amounts Booklet (FAB) that includes visual images of food and standardized shapes. When applicable, research assistants instruct the participants to reference the FAB to help them quantify the portion sizes consumed. The second tool is an ecological momentary assessment (EMA) platform, which captures pre- and post-photographs of eating occasions in real time. Participants are provided with a unique login and receive detailed instructions on how to upload photographs to the platform (which can be accessed via smartphone device) at the Study Visit. They are instructed to upload pre- and post-photographs of all eating occasions during the observation period. Research assistants access the EMA platform, which displays photographs and associated timestamps indicating when an eating occasion occurred. During the 24HR, research assistants reference the uploaded photographs and prompt participants about omitted food or beverage items to aide in quantifying portion sizes consumed.

Data collected from the 24HR are entered into the Nutrition Data System Software for Research (NDSR; Nutrition Coordinating Center, University of Minnesota, Minneapolis, Minnesota) using version 2022. Variables of specific interest include kcal and grams of carbohydrate, protein, and fat (by eating occasion and by day); glycemic index, glycemic load, available carbohydrate, added sugars, and total sugars (by eating occasion and by day); and the Healthy Eating Index (HEI)-2015 (53) (which assigns a continuous, numerical score based on how well the diet aligns with the Dietary Guidelines for Americans).

The timestamps associated with every photo uploaded, pre- and post, are recorded by two independent research assistants; their data files are then cross-checked against each other and the EMA platform for accuracy. Data on the type of eating episode (i.e., breakfast, lunch, snack, dinner/supper, and beverage) are also obtained from the photo uploads, and similarly double entered and crossed-checked. The timestamps obtained from the uploaded photos will be used to identify the pre-breakfast and all postprandial periods (see Section *Glucose outcomes*). If these data are missing, the *Daily Meal Times Survey* data will be used in their place.

#### 2.5.7 Adverse events

No serious adverse events are expected. Potential adverse events include the possibility of skin infection or irritation from the CGM and the risk of injury during the assigned walking or stepping. Adverse events are assessed by participant self-report each morning on the *Daily Sleep Survey* and again, in person by study staff at the Drop-off Visit (when the participants return their equipment and receive their incentives). The trial also has an independent safety officer who advises on the process used to monitor adverse events and is available, as needed, for consultation on the potential need to adjust study procedures for any unanticipated adverse events encountered in the future.

### 2.6 Incentives

Participants receive up to $450 in gift cards to Walmart or Amazon as compensation for their efforts at the conclusion of the trial.

### 2.7 Power and sample size

Due to the efficiency of the randomized crossover design, sample sizes tend to be small. Power calculations for a repeated measures ANOVA focused on the within-subject effect size. Conservatively assuming that the ratio of effect variance to common variance will be low (i.e., 0.15) and that there will be low correlation across measurements (i.e., 0.15) returns an estimated within-subject effect size of f = 0.73. For this within-subject effect size, with a significance level set at .007 (i.e., Bonferroni adjusted for examination of the seven original glucose outcomes) and a nonsphericity correction of 1, the estimated power was 0.89 for a sample size of 36. Since an enrolled participant could be put on insulin or deliver their baby during the observation period, we set out to recruit and randomize 39 participants.

### 2.8 Statistical analyses

Kolmogorov–Smirnov tests will assess normality, and the appropriate repeated measures test employed (e.g., repeated measure ANOVA or Friedmann’s test) to compare glucose outcomes by treatment condition. The primary analysis will be intention to treat; per protocol associations will also be explored. Linear mixed-effects models (i.e., PROC MIXED in SAS) will allow for comparisons of the glucose outcomes across the three treatment conditions with adjustment for GGI or GDM status, BMI, and diet (that is, HEI (53) and/or grams of carbohydrate). Linear mixed-effects models will be additionally adjusted for the volume of PA from activities of daily living during waking hours (or background PA). Modified PSQI (sleep) metrics will also be compared across the three conditions using linear mixed-effects models adjust for GGI or GDM status, sleep duration, number of waking episodes, and PA. Mood states will be compared between the early PA and late PA conditions only, potentially controlling for PA modality (e.g., walking, stepping, or both), circumstances (e.g., while listening to music), and/or intensity (e.g., volume of PA in MET minutes). If needed, multiple imputations will be used to handle missing data.

## 3 Results

All data are expected to be collected by December 2025.

## 4 Discussion

This paper describes the rationale and protocol for the Time to Move Randomized Crossover Trial, which aims to compare the glucose-lowering effects of 30 min of moderate-intensity walking or stepping performed in the morning (i.e., within 30 min to 40 min of starting breakfast) to that performed in the evening (i.e., within 30 min to 40 min of starting after dinner) in pregnant individuals with GGI or GDM. The glucose-lowering effects of PA are well known, despite the many sources of inter- and intra-individual variability that impact the glucose response to a single bout of PA. Given the influence of circadian rhythms on insulin sensitivity (54, 55), the timing of PA may play a role in the glucose response; however, the timing of PA has received little attention in the scientific literature, particularly in the pregnancy literature. As PA is a non-pharmacological, evidence-based strategy for managing glucose levels, leveraging PA timing to improve glucose levels is particularly attractive for individuals with pregnancy hyperglycemia. Indeed, in this population, behavioral counseling for a healthy diet typically recommends a lower carbohydrate intake at breakfast than at lunch and dinner. Behavioral counseling for free-living PA may similarly recommend a preferred time or window of the day to maximize the impact.

The trial’s use of wearable technology, that is, the Dexcom CGM (to assess glucose outcomes) and the ActiGraph (now Ametris) CentrePoint Insight Watch (to assess adherence to PA prescriptions and sleep), is a clear strength. The trial assesses dietary intake via the 24-hour dietary recall method, uniquely augmenting the accuracy of the recall by cross-referencing the interviewee’s responses to photo uploads of all the food and beverages they consumed. To the best of our knowledge, this is the first study to link these three data elements by timestamp, thereby allowing for the investigation of the impacts of the timing of these key health behaviors, as well as their interactions, on the glucose response.

Potential limitations include the possibility that a participant is put on glucose-lowering medication or delivers their neonate during the study period, which would limit and potentially introduce an imbalance in the number of observation days available for analyses per treatment condition. Poor adherence to PA, that is, its prescribed timing, duration, and intensity, could also limit the trial’s findings. Therefore, we carefully vet potential participants at the Information Visits, emphasize the importance of PA, develop a PA Action Plan at the Study Visit, and offer text message reminders for their PA on the days/times it is assigned. Finally, the two-regression algorithms were validated among healthy non-pregnant adults; thus, it is possible that it underestimates PA intensity (METs) in pregnant individuals or late in pregnancy, which could result in a Type 2 error in per-protocol analyses.

This crossover trial will provide valuable insights into whether the timing of postprandial PA, that is, in the morning following breakfast vs. the evening following dinner, differentially impacts glucose levels in individuals with pregnancy hyperglycemia. This investigation will also be the first to provide comprehensive data on the composition and distribution of dietary intake, and sleep in individuals with pregnancy hyperglycemia. We hope that the Time to Move Randomized Crossover Trial will motivate future studies to integrate serial measurements of health behaviors with CGM data and that our findings will lead to a more specific, that is, time of day-based, recommendation for PA in individuals with pregnancy hyperglycemia.
